# The impacts of substance abuse and dependence on neuropsychological functions in a sample of patients from Saudi Arabia

**DOI:** 10.1186/1744-9081-5-48

**Published:** 2009-12-11

**Authors:** Mohamed A Al-Zahrani, Yasser A Elsayed

**Affiliations:** 1 Al-Amal Complex for Mental Health, PO 5054, Dammam 31422, Saudia Arabia; 2 Institute of Psychiatry, Ain Shams University, Abbasia, Cairo, Egypt

## Abstract

**Background:**

A lot of studies were directed to explore the relation between drug abuse and neuropsychological functions. Some studies reported that even after a long duration of disappearance of withdrawal or intoxication symptoms, many patients have obvious deterioration of cognitive functions. The aim of this study was to explore the relationship between the substance use disorders and the executive functions.

**Methods:**

Two groups were selected for this study. An experimental group consisted of 154 patients and further subdivided according to the substance used into three different subgroups: opioid, amphetamine and alcohol groups which included 49, 56 and 49 patients respectively. The control group was selected matching the experimental group in the demographic characteristics and included 100 healthy persons. Tools used were: Benton visual retention tests, color trail making test, Stroop colors-word test, symbol digit modalities test, the five dots cognitive flexibility test, and TAM verbal flexibility test. All the data were subjected to statistical analysis

**Results:**

The study showed that the group of drug-dependent subjects performed significantly worse than the comparison group on all measures Also, there were significant differences among the subgroups as the alcoholic group was much worse followed by the amphetamine then the opioids groups. Patients with longer duration of dependence and multiple hospital readmissions were much worse in comparison to patients with shorter duration of dependence and less readmission.

**Conclusion:**

The study confirmed that the functions of specific brain regions underlying cognitive control are significantly impaired in patients of drug addiction. This impairment was significantly related to type of substance, duration of use and number of hospitalization and may contribute to most of behavioral disturbances found in addicts and need much attention during tailoring of treatment programs.

## Background

A lot of studies were directed to explore the relation between drug abuse and neuropsychological functions. The interest in studying this relationship is the result of the scientific achievements in the field of neuropsychology. Cognitive deficits associated with the chronic abuse of drugs have important theoretical and clinical significance. Such deficits reflect changes to the underlying cortical, sub-cortical and neuromodulatory mechanisms that underpin cognition, and also interfere directly with rehabilitative programs [1,2]. Alcohol induced cognitive deficits include impaired decision-making, response inhibition, planning and working memory [3]. Heroin addicts tended to perform worse than controls particularly on various dimensions of impulsivity, attention, learning, working memory and pattern recognition, all primarily prefrontal functions [4]. Chronic use of amphetamine has been associated with a wide range of cognitive deficits, involving domains of attention, inhibitory control, planning, decision-making, learning and memory [5]. Despite the growing evidence of the negative impacts of different substances of abuse on the brain, neuropsychological research directly comparing cognitive performance in different phases of addiction illness and different substances are still sparse with controversial results.

### Aim of the work

This work was done to test the hypothesis of cognitive dysfunctions associated with chronic use of psychoactive substances in a sample of patients in Saudia Arabia. The study was done to explore the relationship between the substance use disorders and the executive functions and to answer certain important study questions: 1) Are there significant differences between the addicts and healthy people regarding neuropsychological functions? 2) Are there significant differences between users of different substances regarding these neuropsychological functions? 3) Does the duration of abuse lead to significant differences among addicts in the performance of neuropsychological tasks? 4) Does the frequency of hospitalization lead to significant differences among addicts in the performance of neuropsychological tasks?

## Methods

The current study had a cross sectional design. It was done in Al-Amal complex for mental health which is located in Dammam. The complex is affiliated to the ministry of health of the kingdom of Saudia Arabia (KSA). The proposal of the study was approved by both of the scientific and ethical committee of the complex prior to data collection. **Subjects: **To answer the first study question, two groups of subjects were selected, an experimental group consisted of 154 patients and control group consisted of 100 healthy persons. The control group was selected matching the experimental group in the demographic characteristics (age, education level and marital state). To answer the second study question, the patients were further subdivided according to the substance used into three different groups: alcohol, heroin and amphetamine groups which included 49, 49 and 56 patients respectively. The three groups did not differ statistically in age or level of education. These substances were selected because of their popularity in the region and known chronicity. To answer the third study question the patients were also divided into 4 subgroups according to duration of substance use, from 1-5 years, 6-10 years, 11-15 year and >15 years. Lastly, to answer the fourth study question, patients were classified according to the frequency of hospital admission into 4 categories; once, twice, thrice, and ≥ 4 admissions. The patient group was selected from in-patients few days after finishing their detoxification program and according to random list prepared by randomization computer program. Due to limited number of female patients all subjects were males. The exclusion criteria used in this study included any significant use of other substances within past two years and significant history of physical or mental disorders that could impact cognitive functions (eg. mood and anxiety disorders, major organ failure, etc). All subjects were free of withdrawal symptoms and free of drugs at the time of testing. Caffeinated beverages and nicotine were not allowed during the time of testing. **Procedures of the study: **1) Oral informed consent was taken from all subjects 2) Psychiatric assessment including details of substance use and treatment, duration of use and number of hospitalizations. 3) Patients were assessed using mini international neuropsychiatric interview (MINI) [6]. 4) Urine test for different substances of abuse was done for all subjects to confirm the type of substance and exclude other substances 5) Neuropsychological battery to assess cognitive functions. **Tools applied: 1) MINI **which is a short structured diagnostic interview for DSM-IV and ICD-10 psychiatric disorders. The scale was translated to Arabic and validated previously [6] The MINI-Plus was selected over other screening instruments because of its ease of administration. **2) Neuropsychological battery **which was formed from Benton visual retention test, color trail making test (CTMT), Stroop colors-word test, symbol digit modalities test (SDMT), the five dots cognitive flexibility test, TAM verbal flexibility test. All previous tools were translated into Arabic prior to application.

The color trail making test uses numbered colored circles and universal sign language symbols. The circles are printed with vivid pink or yellow backgrounds that are perceptible to colorblind individuals. The test has two forms of application. In the first form, the subject uses a pencil to rapidly connect circles numbered 1 through 25 in sequence. In the second application, the subject rapidly connects numbered circles in sequence, but alternates between pink and yellow colors. It means that the complexity increased in the second form as there are possibilities of both number and color errors. This test is a measure of some of the frontal lobe functions especially cognitive flexibility, perceptual tracking, sequencing of events, sustained and divided attention and graphomotor skills [7].

The Stroop color word task [8] is a test of selective attention and cognitive flexibility. The task takes advantage of our ability to read words more quickly and automatically than we can name colors. If a word is printed or displayed in a color different from the color it actually names we will read the word more readily than we can name the color in which it is displayed. The cognitive mechanism involved in this task is called selective or directed attention, as the subject has to manage his attention, resist interference from irrelevant stimuli, inhibit or stop one response in order to say or do something else. The investigators used the test in 5 forms gradually increasing in complexity. In the first form, the subject was asked to recognize colors. In the second form, the subject was asked to read the words written in black (names of colors). In the third form, the subject was asked to read the names of the colors printed in different colors. In the fourth form, the subject was asked to recognize the color of the printed words (names of colors). In the last form, the subject was asked to serially read the words in one row and recognize the color in one row. The time needed to each application and number of errors was recorded on a special form.

The symbol digit modalities test (SDMT) [9] involves a simple substitution task that normal children and adults can easily perform. Using a reference key, the examinee has 90 seconds to pair specific numbers with given geometric figures. This test is a measure of speed of mental processing, attention and concentration, divided attention, ability to fix and detect errors and learn from them. The scoring ranges from 0-110, with higher scores representing better performance.

The five dots test assesses figural fluency and cognitive flexibility. Figural fluency is considered one of the executive functions of the right frontal lobe. Patients are instructed to connect standardized 5 points to do the largest number of different designs within 5 minutes [10].

TAM verbal flexibility is a test of word fluency that considered an executive function for the left frontal lobe. It is considered an Arabic analogue of FAS controlled oral word association test [8,11].

The Benton revised visual retention test [12] is a widely used instrument that assesses visual-motor coordination, visual perception, visual memory, and attention and visuoconstructive abilities. It was translated to Arabic and validated previously [13]. In this study the investigators used the test in three forms, in the first form each subject was asked to copy a shape while seeing it. In the second form each subject was asked to copy a shape from the memory immediately after seeing it for 5 seconds and in the last form each subject was asked to copy a shape from the memory 10 seconds after removal of the shape.

### Statistical Analysis

All the data was subjected to statistical analysis using SPSS version 10.0. Tests for comparisons used included the mean and standard deviation and multivariate analysis of co-variance (MANCOVA). Level of significance was detected at p value 0.05.

## Results

### Patient control comparisons

The mean age of patient group was 33.16 ± 8.3 years and the mean age for the control group was 31.24 ± 9.4 with no statistical significant differences between. All the age ranges and education are showed in Table 1.

**Table 1:** Age and level of education of patient and control groups

	Number of patients according to years of education					Mean age
Group	≤ 6 years	7-9 years	10-12 years	> 12 years	Age range	± SD
Alcohol	7	16	22	5	19-54	30.22 ± 8.35

Heroin	7	15	23	5	21-51	32.51 ± 9.22

Amphetamine	8	14	28	4	18-49	33.34 ± 7.14

All patients	22	45	73	14	18-54	33.16 ± 8.3

Control	14	29	49	8	18-50	31.24 ± 9.4

**As shown in Table **2, **in color trail making test (CTMT), **there were statistically significant differences between patients and control groups as the time needed by patient group was more in form 1 and 2. Also, the patient group had more color sequence errors in the first form and more color and number sequence errors in the second form.

**In Stroop task, there was **no significant difference in form 1 while in form 2 and 3 there were statistically significant differences only in time needed and p value was 0.05 (see Table 3). Also in Stroop form 4 and 5, there were statistically significant differences as regard to time needed and number errors.

As shown in Table 4, the results of **five dots test **denoted that the total score of the test was better with control group than with patient group while the patient group revealed more repetitions and less number of unique designs.

**Table 2:** Results of the color trail making test (CTMT)

CTMT	Item	Patients mean ± SD	Control mean ± SD	F value	P value
Form 1	Time	74.72 ± 1.95	68.26 ± 2.14	F(1,15) = 4.52	0.05
	
	Color errors	0.71 ± 0.08	0.28 ± 0.09	F(2,16) = 10.92	0.001

Form 2	Time	118.36 ± 3.01	105.60 ± 3.30	F(1,40) = 7.37	0.01
	
	Number errors	0.47 ± 0.08	0.17 ± 0.09	F(2,20) = 5.95	0.01
	
	Color errors	0.75 ± 0.09	0.33 ± 0.10	F(3,21) = 8.03	0.005

CTMT: color trail making test.

Higher scores indicate poorer function

**Table 3:** Results of Stroop colors-word test

Stroop	Patients Mean ± SD	Control Mean ± SD	F value	P value
Time 2	17.92 ± 0.49	16.49 ± 0.54	F(1,331) = 3.87	0.05

Time 3	13.63 ± 0.26	12.77 ± 0.29	F(1,16) = 4.50	0.05

Time 4	26.53 ± 0.68	25.00 ± 0.75	F(1,98) = 3.92	0.05

Time 5	30.15 ± 0.69	26.18 ± 0.76	F(1,22) = 13.45	0.001

Errors 4	2.70 ± 0.16	1.50 ± 0.18	F(1,9) = 22.46	0.001

Errors 5	2.63 ± 0.21	2.19 ± 0.23	F(1,110) = 3.89	0.05

Higher scores indicate poorer function

**Table 4:** Results of five dots, TAM verbal flexibility and symbol digit modalities tests (SDMT)

Test	Items	Patients mean ± SD	Control mean ± SD	F value	P value
Five dots test	Total score^a^	27.92 ± 0.63	33.81 ± 0.69	F(1,6) = 35.87	0.001
	
	Unique designs^a^	24.63 ± 0.60	31.50 ± 0.66	F(1,4) = 54.27	0.001
	
	Repetitions ^b^	3.38 ± 0.28	2.58 ± 0.31	F(1,161) = 3.90	0.05

TAM test	Words ^a^	34.69 ± 1.25	45.25 ± 1.38	F(1,7) = 29.11	0.001
	
	Interference ^a^	1.02 ± 0.15	0.25 ± 0.16	F(1,110) = 11.04	0.001
	
	Repetitions ^b^	0.69 ± 0.09	0.18 ± 0.10	F(1,35) = 12.90	0.001
	
	Total score ^a^	41.90 ± 1.18	51.79 ± 1.29	F(1,7) = 29.04	0.001

	SDMT ^a^	30.10 ± 1.43	54.42 ± 1.58	F(1,110) = 117.13	0.000

a. Higher scores indicate better function.

b. higher scores indicate poorer function

TAM: TAM verbal flexibility test.

SDMT: Symbol digit modality test

Also the total score of **TAM verbal flexibility test **was better with control group than patient group as they succeeded in naming of larger numbers of words with less repetition and interference than patient group. The **scores of symbol digit modalities test (SDMT) **were higher for the control group denoting better functions.

On the Benton visual retention test, control subjects scored significantly better and had significantly fewer errors than did drug-dependent patients (see Table 5).

**Table 5:** Results of Benton visual retention test

Benton	Item	Patients mean ± SD	Control mean ± SD	F value	P value
Form 1	Correct Responses ^a^	5.76 ± 0.18	6.43 ± 0.20	F(1,7) = 5.68	0.05
	
	Errors ^b^	5.32 ± 0.2	4.45 ± 0.27	F(2,43) = 5.15	0.01

Form 2	Correct responses ^a^	6.30 ± 0.19	6.90 ± 0.21	F(1,50) = 4.03	0.05
	
	Errors ^b^	3.92 ± 0.21	3.23 ± 0.23	F(1,14) = 4.60	0.05

Form 3	Correct responses ^a^	6.56 ± 0.17	7.10 ± 0.18	F(1,30) = 4.17	0.05
	
	Errors ^b^	3.42 ± 0.18	2.79 ± 0.20	F(2,44) = 5.12	0.01

a. Higher scores indicate better function.

b. higher scores indicate poorer function

### Differences between patients according to type of substance

As shown in Table 6, the alcoholic group took longer time than the heroin and amphetamine groups to achieve the responses in the first form of color trail making test (**CTMT) **while in the second form the worst was the amphetamine group followed by alcohol and the best was the heroin group. In the second form of the test, the number of errors with alcoholic group was significantly higher than with heroin and lastly amphetamine groups. **In Stroop test, **there were statistically significant differences between the three groups of patients as the time needed in all forms of the test was significantly more with alcoholic groups followed by amphetamine group and lastly the heroin group. Also, the number of errors was more with the alcoholic group in form number 1 and 2 while it was more with amphetamine group in form number 5. **In five dots test, **there were statistically significant differences between the three patients groups as the total score of the test and the ability to produce unique designs were better with heroin group followed by alcohol and lastly the amphetamine.

**Table 6:** Differences between patients according to type of substance

Test items	Alcohol mean ± SD	Heroin mean ± SD	Amphetamine mean ± SD	F value	P value
CTMT Time 1 ^b^	85.69 ± 34.14	75.3 ± 22.09	69.47 ± 18.61	F(2,101) = 5.3	0.006

CTMT Time 2 ^b^	120.37 ± 41	111.34 ± 33.42	132.62 ± 5.02	F(2,101) = 5.02	0.008

CTMT errors 2 ^b^	0.59 ± 1.25	0.27 ± 0.84	0.19 ± 1.33	F(2,62) = 3.18	0.048

Stroop Time 4 ^b^	31.92 ± 6.92	24.95 ± 7.84	25.78 ± 6.13	F(1,101) = 14.78	0.000

Stroop Time 5 ^b^	36.28 ± 8.07	28.29 ± 9.48	29.96 ± 8.40	F(1,101) = 11.94	0.000

Stroop Errors 5 ^b^	2.95 ± 2.86	2.36 ± 2.20	3.59 ± 3.25	F(3,35) = 2.90	0.05

5 dots total score ^a^	26.08 ± 6.84	30.16 ± 9.29	24.88 ± 7.81	F(5,85) = 3.24	0.01

5 dots Unique designs ^a^	22.57 ± 6.71	27.45 ± 8.44	21.33 ± 7.18	F(4,101) = 3.49	0.01

5 dots Repetitions ^b^	3.66 ± 4.11	2.79 ± 2.74	3.7 ± 3.2	F(5,85) = 3.24	0.01

TAM interference ^b^	0.8 ± 2.01	1.6 ± 2.61	0.53 ± 1.5	F(4,101) = 3.49	0.01

TAM repetitions ^b^	0.38 ± 0.8	1.18 ± 1.53	0.43 ± 1.29	F(3,38) = 6.66	0.001

TAM words ^a^	33.25 ± 12.59	36.28 ± 16.6	33.85 ± 15.45	F(7,101) = 2.07	0.05

TAM total score ^a^	40 ± 11.78	42.32 ± 16.59	41.13 ± 14.73	F(13,166) = 1.76	0.05

SDMT ^a^	27.04 ± 10.02	32.8 ± 10.88	26.89 ± 10.11	F(4,42) = 5.64	0.001

a. Higher scores indicate better function.

b. higher scores indicate poorer function

CTMT: color trail making test

TAM: TAM verbal flexibility test.

SDMT: symbol digit modalities test

**In TAM verbal flexibility test and symbol digit modalities test (SDMT)**, there were statistically significant differences between groups as the scores were more in the heroin group in all aspects of the test followed by the other two groups. Lastly in **Benton test, **there were no statistically significant differences between the different subgroups of patients in all forms of the test.

### Differences between patients according to duration of use

As shown from Table 7, in color trail making test (CTMT), Stroop test, five dots test and symbol digit modalities test (SDMT), there were statistically significant differences between the different durations as the group of the least duration (1-5 years) was relatively the best group in performance and the group of the longest duration(> 15 years) was the worst. The same was also noticed as regard rotation errors in Benton which increased significantly with the duration of use. However, there were no statistically significant differences between the groups in TAM verbal flexibility test and other aspects of Benton test. The number of color errors in color trail making test (CTMT) was more with the group of the shortest duration.

**Table 7:** Differences between patients according to duration of use

	Duration of substance use					
Tests used	1-5 Years mean ± SD	6-10 Years mean ± SD	11-15 Years mean ± SD	>15 Years mean ± SD	F value	P value
CTMT Time 1 ^b^	70.68 ± 32.55	71.92 ± 25.81	75.60 ± 19.73	85.39 ± 22.61	F(3,101) = 2.74	0.05

CTMT Color errors 1 ^b^	1.11 ± 1.41	0.45 ± 0.96	0.31 ± 0.86	0.84 ± 1.19	F(4,45) = 3.75	0.01

CTMT Time 2 ^b^	115.63 ± 34.03	119.04 ± 29.24	122.49 ± 31.82	133.15 ± 40.95	F(3,80) = 2.83	0.04

Stroop time 4 ^b^	25.38 ± 6.36	26.06 ± 6.61	28.87 ± 8.66	29.31 ± 8.28	F(3,101) = 2.68	0.05

Stroop Errors 4 ^b^	2.76 ± 2.37	2.50 ± 2.00	3.29 ± 1.78	3.73 ± 2.46	F(3,50) = 2.80	0.05

Stroop Time 5 ^b^	29.04 ± 7.77	29.71 ± 7.88	31.93 ± 10.15	34.35 ± 10.41	F(7,80) = 2.87	0.01

5 dots total score ^a^	29.51 ± 7.98	28.45 ± 7.89	26.47 ± 2.94	24.64 ± 7.96	F(7,100) = 2.81	0.01

5 dots unique designs ^a^	26.16 ± 7.99	25.28 ± 7.81	22.57 ± 8.76	21.93 ± 6.94	F(3,101) = 2.70	0.05

SDMT ^a^	28.95 ± 11.16	31.85 ± 9.65	30.88 ± 10.58	25.49 ± 10.55	F(4,35) = 3.01	0.032

Benton Rotation errors ^b^	0.32 ± 0.53	0.63 ± 0.87	1.22 ± 1.41	0.87 ± 1.24	F(2,81) = 4.452	0.01

a. Higher scores indicate better function.

b. higher scores indicate poorer function

CTMT: color trail making test

SDMT: symbol digit modality test

### Differences between patients according to number of hospitalizations

In all tests applied, the scores were better in the group admitted once and worst in the group of ≥ 4 admissions (see Table 8 and 9). In the first application of Benton (Table 9), the total number of errors, the distortion errors and the rotation errors were less in the group of least admission and increased gradually with increase of admissions and p value ranged from 0.05 to 0.01. In the second application the number of correct answers decreased significantly with the increase in number of admissions and p value persisted at the same level 0.01. While in the third application, the number of errors, the number of correct answers and the distortions were significantly worse with the group of ≥ 4 admissions.

**Table 8:** Differences between patients according to number of hospitalizations: color trail making (CTMT), Stroop and five dots tests

	Number of hospitalizations (n)					
Tests used	1 (44) mean ± SD	2 (41) mean ± SD	3 (38) mean ± SD	≥ 4 (31) mean ± SD	F value	P value
CTMT Time 1 ^b^	67.40 ± 15.80	68.96 ± 19.14	76.28 ± 21.12	99.59 ± 37.42	F(1,98) = 13.413	0.000

CTMT Color errors 1 ^b^	0.50 ± 0.98	0.39 ± 0.86	0.68 ± 1.16	1.39 ± 1.48	F(4,50) = 5.457	0.001

CTMT Time 2 ^b^	103.52 ± 23.34	114.82 ± 30.57	123.74 ± 29.48	150.55 ± 42.84	F(1,45) = 14.309	0.000
CTMT number errors 2^b^	0.32 ± 0.80	0.15 ± 0.57	0.45 ± 1.16	1.16 ± 1.77	F(4,48) = 5.465	0.001

CTMT Color errors 2 ^b^	0.48 ± 1.11	0.68 ± 1.46	0.83 ± 1.15	1.35 ± 1.60	F(7,76) = 2.861	0.01

Stroop Time 1 ^b^	10.40 ± 2.21	11.65 ± 1.90	12.08 ± 2.31	15.13 ± 6.06	F(1,265) = 12.662	0.001

Stroop Errors 1 ^b^	0.07 ± 0.33	0.05 ± 0.22	0.08 ± 0.36	0.33 ± 0.83	F(1,77) = 2.706	0.05

Stroop Time 5 ^b^	26.31 ± 7.87	29.42 ± 7.53	31.87 ± 8.04	40.49 ± 8.31	F(1,10) = 20.611	0.001

Stroop Errors 5 ^b^	2.02 ± 2.22	2.87 ± 3.22	3.39 ± 2.54	3.86 ± 2.96	F(1,265) = 12.662	0.001

Total score 5 dots ^a^	31.48 ± 7.73	26.37 ± 8.51	25.76 ± 7.26	23.90 ± 8.31	F(3,39) = 6.587	0.001

5 dots unique designs ^a^	27.93 ± 7.81	23.68 ± 7.64	22.26 ± 7.09	20.71 ± 7.57	F(3,37) = 6.638	0.001

n. number of patients

a. Higher scores indicate better function.

b. higher scores indicate poorer function

CTMT: color trail making test

**Table 9:** Differences between patients according to number of hospitalizations: Tam verbal flexibility, symbol digit modalities (SDMT) and Benton visual retention tests

	Number of hospitalizations (n)					
Tests Used	1 (44) mean ± SD	2 (41) mean ± SD	3 (38) mean ± SD	≥ **4** (31) mean ± SD	F value	P value
TAM correct words ^a^	43.39 ± 16.02	34.73 ± 12.21	29.42 ± 11.55	25.27 ± 11.34	F(1,28) = 13.682	0.001

TAM score ^a^	49.50 ± 16.40	42.46 ± 12.74	36.76 ± 10.63	33.17 ± 10.92	F(1,121) = 11.321	0.001

SDMT ^a^	31.05 ± 8.69	30.56 ± 10.97	29.71 ± 11.59	23.61 ± 10.38	F(4,61) = 3.693	0.01

Benton Errors 1 ^b^	4.84 ± 2.11	5.15 ± 2.89	5.74 ± 2.63	6.55 ± 2.88	F(6,132) = 2.927	0.01

Benton Distortions 1 ^b^	2.93 ± 1.76	2.93 ± 2.04	3.45 ± 2.13	4.10 ± 2.10	F(3,118) = 2.682	0.05

Benton Rotation errors 1 ^b^	0.48 ± 0.70	0.83 ± 0.95	1.08 ± 1.22	0.87 ± 0.99	F(3,74) = 2.741	0.05

Benton responses 2 ^a^	6.55 ± 2.24	6.59 ± 2.16	6.08 ± 2.21	5.19 ± 2.33	F(7,76) = 2.898	0.01

Benton responses3 ^a^	6.48 ± 1.97	6.37 ± 1.61	7.27 ± 1.84	5.65 ± 1.87	F(5,76) = 4.535	0.001

Benton errors 3 ^b^	3.50 ± 1.94	3.66 ± 1.62	2.71 ± 1.81	4.29 ± 1.87	F(3,101) = 4.475	0.005

Benton Distortions 3 ^b^	2.80 ± 2.15	3.24 ± 2.05	2.24 ± 2.16	4.00 ± 1.95	F(3,143) = 4.388	0.005

n. number of patients

a. Higher scores indicate better function.

b. higher scores indicate poorer function

TAM: TAM verbal flexibility test.

SDMT: symbol digit modality test

## Discussion

Several authors have stressed the presence of cognitive deficits in patients with substance use disorders however most of studies focused on alcohol [14-16]. The current study tried to test the hypothesis of cognitive dysfunctions associated with three common substances of abuse in Saudia Arabia and tried to find the effect of different substances and the relation of cognitive deficits to duration of use and number of hospitalizations. It described an assessment of neuropsychological functions in newly abstinent drug dependent subjects and in healthy non-drug using controls. The results showed that the group of drug-dependent subjects performed significantly worse than the comparison group on all measures. The differences increased much with the increase in the complexities of the tasks. Additionally, significant differences in neuropsychological function were identified in groups based on primary drug of abuse. Also, the study reported significant differences in neuropsychological functions in groups based on duration of addiction and number of hospitalizations, with subjects who report a shorter duration of addiction and less frequent hospitalization generally out-performing subjects with longer periods of addiction and more frequent hospitalization. All these findings confirmed the presence of impairment in the functions of frontal and temporal lobes which considered responsible for the cognitive flexibility and may be one of the reasons behind failure of some patients to change their life style.

Impaired color trail making test (CTMT) reflects disturbance of cognitive flexibility, perceptual tracking, sequencing of events, sustained and divided attention and graphomotor skills. This test denoted disturbed functions of lateral temporal lobe which is the brain area responsible for reception of stimuli, reduction of irrelevant stimuli, sending relevant information to frontal lobe to start to achieve [7]. Moreover, the results of this test behaviorally confirmed that patients may have significant impairments in their abilities to; track any task to the end, arrange events, maintain attention and resist irrelevant stimuli. This finding is one of the important factors behind failure of patients to maintain abstinence, marriage, study or work.

The results of Stroop test indicated that the differences increased gradually with the different applications of the test and reach its maximum in the fourth and fifth application where p value was highly significant (0.001). These results confirmed that the patients had impaired selective attention, concept formation, correction of errors, set shifting, behavioral control and modifications according to stimuli, inhibition of irrelevant responses, self-regulation capability and cognitive flexibility more than the control group. This may explain the inability of some patients to shift rapidly and adequately from one behavior to another. Although they know the dangerousness of their behaviors, they cannot control or change them and persist on the same style [17,18]. Impaired performance on Stroop test reflects impaired activation of different brain areas including the anterior cingulate cortex, dorsolateral prefrontal cortex, parietal lobule, insula, and striatum [19]. This deficit may contribute to the impulsivity seen in patients with substance use disorders especially in response to substance related cues. Also, it may explain the frequent relapses of patients [20].

The results of five dots test indicated that the patients had significant impairment of figural fluency, innovation ability and cognitive flexibility more than the control group. The results of this test reflect impairment of frontal lobe functions and may contribute to the ritualistic stereotyped behaviors found in addicts [21].

Not only the figural fluency but also word fluency was significantly impaired in patients as revealed by TAM verbal flexibility test. Impairment of TAM test indicates dysfunction of the left frontal lobe and may contribute to disturbed use of language by patients. That's why some patients with chronic substance use tend to be socially isolated and cannot make new friends as they cannot communicate effectively through language [22].

The speed of mental processing and the ability of patients to fix and detect errors and learn from them were significantly impaired as indicated from symbol digit modalities test (SDMT). That's why some patients continued to use the substance whatever the consequences and suffered from several relapses due to the same mistakes in addition to slowing of mental processing that impair their abilities to take the proper decisions at the proper time [23].

Lastly, the patients suffered from significant impairment of visual-motor coordination, visual perception, visual memory and visuoconstructive abilities as indicated by results of Benton visual retention test. Also, this test reflects impairment of non-dominant temporal and occipital lobes as well as dorsolateral prefrontal cortex [24] which is known to modulate problem solving, spatial planning and corresponds with performance problems found in the present study. The poor performance of patient group on Benton test supported the findings of other studies and suggested a presence of structural damage to the hippocampus out of the toxic effect of the substances [25].

A lot of other studies observed that there is a strong relation between abuse of substances and executive functions especially cognitive flexibility and attention. Most of these studies focused on alcohol [26-28]. The same results were also attained in cocaine [29,30], heroin [22] and amphetamine users [31]. All these studies indicated a significant relationship between addiction and deterioration of executive functions although different tests and study protocols were used. However, the results in the current study are opposing to results of Rounsavillae et al study [32] who reported that neuropsychological functions in a sample of opiate users were better than the control groups. However, in this study the control group was mainly a group of epileptic patients. In Qassem et al [22], similar results were found as regard presence of cognitive dysfunctions in a group of heroin users but in this study they used Luria Nebraska battery and addiction severity index not only duration of use and number of hospitalization as in the current study.

Some studies found that the impairment of cognitive functions is related to the amount and concentration of the substance especially alcohol [33,34]. It was difficult in the current study to include such information as alcohol is prohibited legally and the available alcohol in this region is poorly synthesized and has unknown concentration. Also, the amphetamine available is poorly synthesized with a lot of adulterations and differences in concentration of the active material within the tablets [35]. The same was also true as regard heroin as many patients don't suffer from withdrawal symptoms due to substance adulteration and the dose doesn't indicate accurately the concentration of substance [36].

In Table 6 by comparing the three subgroups of patients, it was found that the alcoholic group was much worse than amphetamine and lastly heroin group on most of aspects. Although, the neuropsychological dysfunctions associated with alcohol are well studied and documented through a lot of studies [15,27,37], the studies that directly compare the neuropsychological effects of other substances with each other are sparse. Similar to the current study alcoholics were much worse in Robinson et al study [38] but the comparison subjects were cocaine addicts. Also, in another study alcoholics were much worse than heroin users [39]. Some other studies focused more on comparisons of single substance users versus polydrug users as in Bulla et al study [29]and Rosselli and Ardilla study [30] in which they found that the concomitant use of cocaine plus alcohol or other substances have additive negative effects on the brain as compared to the use of only one substance. In Lawton et al study [16], users of stimulants were compared to users of both alcohol and stimulants and it was found that both groups has negative neuropsychological impacts with more impairments in heroin plus amphetamine group. It looks sound that use of more than one substance has more negative additive effect on the cognitive functions that's why the current study didn't consider including a group of polysubstance users as a priority.

It is clear that Benton test didn't differentiate between users of different substances although it differentiates between patient and control groups. That's because the task in Benton test primarily depends on attention and it seems that all substances impact attention negatively.

Following the alcohol group, it was the amphetamine group that showed more negative impacts on different aspects of cognitive functions and this was in accordance with some other studies [29,38,40]. However, in most of these studies, the subjects were cocaine users. Both amphetamine and cocaine have nearly similar effects on the brain. One reason for this significant competition of amphetamine with the alcohol is the findings from a Saudi study [35] in which the structure of the available amphetamine in Saudia Arabia revealed adulteration with a lot of more dangerous substances like ephedrine, arsenic and mercury.

The current study chose the duration of use and number of hospitalizations as indicators for severity of substance use problem. All the results of the comparison of different durations of use by different tests revealed that the increase in duration of use is always associated with more deterioration of cognitive dysfunctions. This finding was in accordance with Becker et al study [33] in which alcoholic patients developed presenile deterioration of cognitive functions.

Interestingly, impairments were found and different from control group even in the group of shortest duration of dependency (1-5 y). It seems that the different substances have relatively rapid neurotoxic effects, including alterations in grey and white matter, structure and function of the hippocampus, amygdala, nucleus accumbens, anterior cingulate cortex, orbito-frontal cortex, and perturbations in neurotransmitter responses and metabolism throughout the mesocorticolimbic system [41,42].

Also the increase in number of hospitalizations was associated significantly with more cognitive deficits. This finding was the same on all neuropsychological tests used. These results are suspected as the number of hospitalizations is directly related to severity of addiction, number of relapses, duration of use, and impairment in cognitive dysfunctions.

Impairment of cognitive functions of patients may be an important factor affecting the outcome of treatment especially that the main treatment in substance abuse is cognitive behavioral therapy. Those patients need different treatment programs to improve their attention and cognitive flexibility. There is a need for rethink the way in which substance users are assessed at the start of any treatment program as they need assessment for their cognitive functions through the different neuropsychological batteries. Also, psychiatrists should consider the different effects of different substances on cognitive functions of patients. Moreover, the duration of any treatment program should be tailored to patients needs as patients with cognitive impairment need longer durations in a protective environment.

In future studies correlation of neuropsychological testing results with functional brain imaging is highly recommended. Moreover, to know the exact relation of this cognitive dysfunction associated with substances and whether it is a trait, state or scar marker, it is beneficial to do longitudinal studies and to take patients in different stages of addiction and abstinence.

### Limitations

A common problem in most of substance use studies is the difficulty to find patients with single substance of abuse that's why the recruitment of cases in the current study took 18 months. Also, matching of patients and control then matching of patient subgroups was a difficult task and many patients were excluded to allow this matching. Another limitation is that patients were tested at few days post-detoxification, limiting the ability to generalize to longer periods of abstinence. It is important to note that most studies of substance use disorders suggest that drug use affects cognitive function even though baseline measures are not available but some level of impairment may antedate and perhaps contribute to the development of drug abuse particularly in light of evidence that cognitive dysfunction is more prevalent in adolescents who are at high risk for substance abuse [4,43].

## Conclusion

The current study suggests that the functions of specific brain regions underlying cognitive control are significantly impaired in patients of drug addiction. This impairment was identified in groups based on primary drug of abuse, duration of addiction and number of hospitalizations. Cognitive dysfunctions may contribute to most of behavioral disturbances found in patients with substance use disorders and need much attention during tailoring of treatment programs for patients.

## Competing interests

The authors declare that they have no competing interests.

## Authors' contributions

MAZ conceived of the study, put the study design, and participated in data collection, application of instruments and statistical analysis. YAE participated in study design, data collection and application of all instruments in addition to interpretation of results and writing of the manuscript. Both authors read and approved the final manuscript.
